# Increased respiratory modulation of cardiovascular control reflects improved blood pressure regulation in pregnancy

**DOI:** 10.3389/fphys.2023.1070368

**Published:** 2023-03-21

**Authors:** Martín Miranda Hurtado, Craig D. Steinback, Margie H. Davenport, Maria Rodriguez-Fernandez

**Affiliations:** ^1^ Institute for Biological and Medical Engineering, Schools of Engineering, Medicine and Biological Sciences, Pontificia Universidad Católica de Chile, Santiago, Chile; ^2^ Neurovascular Health Laboratory, Faculty of Kinesiology, Sport and Recreation, University of Alberta, Edmonton, AB, Canada; ^3^ Program for Pregnancy and Postpartum Health, Physical Activity and Diabetes Laboratory, Faculty of Kinesiology, Sport and Recreation, University of Alberta, Edmonton, AB, Canada

**Keywords:** pregnancy, hypertension, synchronization, wavelet transform, wavelet phase coherence

## Abstract

Hypertensive pregnancy disorders put the maternal-fetal dyad at risk and are one of the leading causes of morbidity and mortality during pregnancy. Multiple efforts have been made to understand the physiological mechanisms behind changes in blood pressure. Still, to date, no study has focused on analyzing the dynamics of the interactions between the systems involved in blood pressure control. In this work, we aim to address this question by evaluating the phase coherence between different signals using wavelet phase coherence. Electrocardiogram, continuous blood pressure, electrocardiogram-derived respiration, and muscle sympathetic nerve activity signals were obtained from ten normotensive pregnant women, ten normotensive non-pregnant women, and ten pregnant women with preeclampsia during rest and cold pressor test. At rest, normotensive pregnant women showed higher phase coherence in the high-frequency band (0.15-0.4 Hz) between muscle sympathetic nerve activity and the RR interval, blood pressure, and respiration compared to non-pregnant normotensive women. Although normotensive pregnant women showed no phase coherence differences with respect to hypertensive pregnant women at rest, higher phase coherence between the same pairs of variables was found during the cold pressor test. These results suggest that, in addition to the increased sympathetic tone of normotensive pregnant women widely described in the existing literature, there is an increase in cardiac parasympathetic modulation and respiratory-driven modulation of muscle sympathetic nerve activity and blood pressure that could compensate sympathetic increase and make blood pressure control more efficient to maintain it in normal ranges. Moreover, blunted modulation could prevent its buffer effect and produce an increase in blood pressure levels, as observed in the hypertensive women in this study. This initial exploration of cardiorespiratory coupling in pregnancy opens the opportunity to follow up on more in-depth analyses and determine causal influences.

## 1 Introduction

Hypertensive pregnancy disorders lead to significant maternal-fetal morbidity and mortality worldwide (Yousif et al., 2019). Increased blood pressure (BP) above 140/90 mmHg after 20 weeks of gestation is defined as gestational hypertension (Espinoza et al., 2019). Preeclampsia is characterized by high BP together with proteinuria (24-h urine protein >0.3 g/day or protein/creatinine ratio ≥0.3) or at least one of the following: thrombocytopenia, renal failure, impaired liver function, pulmonary edema, and visual symptoms (Lakhno, 2017). Despite the high incidence of these pathologies, the mechanisms by which hemodynamic imbalance occurs are unclear (Reyes et al., 2018). Understanding how the cardiovascular, nervous, and respiratory systems relate to each other from a dynamic point of view in healthy and hypertensive pregnancy can provide relevant insights into adaptative changes associated with blood pressure regulation.

From the cardiovascular point of view, BP decreases in the first 8 weeks of pregnancy due to the release of nitric oxide, which decreases peripheral vascular resistance and, as a consequence, decreases preload and increases cardiac output and heart rate (Meah et al., 2016). At the beginning of the third trimester, BP begins to increase, and increased arterial compliance is essential to accommodate the more than 150% increase in plasma volume (Chapman et al., 1998). From the nervous system point of view, sympathetic tone activity increases between 50% and 150% from the sixth week of gestation and remains high during the second and third trimesters (Jarvis et al., 2012). Because of this, pregnancy is considered a healthy state of sympathoexcitation (Usselman et al., 2015a). However, this increase in sympathetic activity does not usually translate into increased BP in what seems to be disrupted transduction between muscle sympathetic nerve activity (MSNA) and peripheral resistance (Usselman et al., 2015b). On the contrary, in women with hypertensive pregnancy disorders, the increase in sympathoexcitation is accompanied by elevations in BP (Spradley, 2019). The damping effect of the increase in sympathetic tone has been related to the concomitant increase in nitric oxide (NO), which could alter signal transduction and sympathetic responsiveness (Cockell and Poston, 1997; Williams et al., 1997). A failure in this poorly understood mechanism could cause elevated BP in hypertensive pregnant women. Kuzhanthaivelu et al. (Karthiga et al., 2021) reported a significant correlation between decreased NO levels and cardiovagal modulation using spectral analysis of heart rate variability. Therefore, hypertensive pregnancy disorders seem to reflect disruptions in the interaction of systems related to BP control that can be studied from a dynamic approach. Methods to study this phenomenon in terms of amplitude and frequency are limited in assessing causal mechanisms that may underlie systemic behavior (Lucchini et al., 2018).

Synchronization analysis allows evaluation of the interdependence of signals obtained from peripheral nerves, respiration and cardiovascular variables (Mcclintock and Stefanovska, 2003) to understand how these processes are affected by pathology. One way to apply this approach is through the relationship between the signals’ phases (Federation, 2001) to characterize the dynamic alterations of control mechanisms during transition processes (Negulyaev et al., 2019). Previous studies (Karavaev et al., 2009; Ocon et al., 2009; Lackner et al., 2011; Ocon et al., 2011; Zhang et al., 2015; Negulyaev et al., 2019; Negulyaev et al., 2021) have analyzed phase synchronization between cardiovascular and respiratory systems in different contexts, including the course of a healthy pregnancy (Moertl et al., 2013). However, they use the Hilbert Transform without considering the multifrequency nature of cardiovascular signals, so estimating instantaneous phases becomes inappropriate. High coherence can be interpreted as phase locking, a requisite for defining phase synchronization (Woisetschläger et al., 2000). Wavelet phase coherence (WPCO) is a more suitable method for evaluating the dynamic interaction of the system because it: i) adapts better to the non-linear behavior of the systems; ii) provides additional information for signals with multiple frequency components giving the degree of coherence at each frequency; iii) allows to study causality between the processes; iv) gives a quantitative measurement of the coupling degree of the systems under study (Woisetschläger et al., 2000).

The cold pressor test (CPT) allows the evaluation of the cardiovascular control by the autonomic nervous system, causing an increase in the sympathetic output that leads to an increase in BP and HR. Hubli et al. (Hubli et al., 2018) described the mechanism behind the biphasic response of BP to CPT in healthy individuals. The first phase of the response is due to the increase in sympathetic tone associated with local pain and temperature receptors, which produces an increase in BP not mediated by baroreceptors. The second phase shows the ability of the system to compensate for the rise in pressure by means of baroreflex control mechanisms, that is, an increase in cardiac parasympathetic tone and a decrease in sympathetic vascular tone. This clinical test was used in pregnancy studies showing that normotensive pregnant women have a blunted response while women with preeclampsia developed an elevated response (Woisetschläger et al., 2000). Therefore, it has been suggested as a screening tool for pregnancy-related hypertensive disorders. Besides being a simple tool, CPT provides information on complex cardiovascular regulation mechanisms. Hypertensive disorders in pregnancy hide functional autonomic disorders that can be expressed as a disruption of the relationship between systems. Baroreflex and neurovascular transduction study the problem locally and do not quantify the causal relationship between BP and HR variability. Therefore, CPT was used here to study how the system responds to a cardiovascular stressor involving an increase in the sympathetic system.

To the best of our knowledge, this is the first study to evaluate differences in phase coherence of cardiovascular, nervous, and respiratory fluctuations in normotensive (NP), hypertensive (HYP), and normotensive non-pregnant (NNP) women at rest and during reflex sympathetic activation in high frequency (HF, 0.15-0.4 Hz) and low frequency (LF, 0.04–0.15) bands. Continuous wavelet transform was used for phase extraction, and WPCO was calculated to evaluate the phase coherence of R-R intervals (RR), BP, MSNA, and respiration. We evaluated the response at rest and CPT to provide quantitative indices of the mechanisms behind optimal blood pressure regulation.

## 2 Methods

### 2.1 Protocol

Data from ten women with preeclampsia, ten normotensive pregnant women, and ten normotensive non-pregnant women obtained from a previous protocol (Yousif et al., 2019) were used for this study. Pregnant participants had singleton pregnancies and were tested in the third trimester. All test protocols were approved by the Health Research Ethics Board at the University of Alberta (Pro00041144) and conformed to institutional guidelines and the standards set by the latest revision of the Declaration of Helsinki.

**FIGURE 1:**
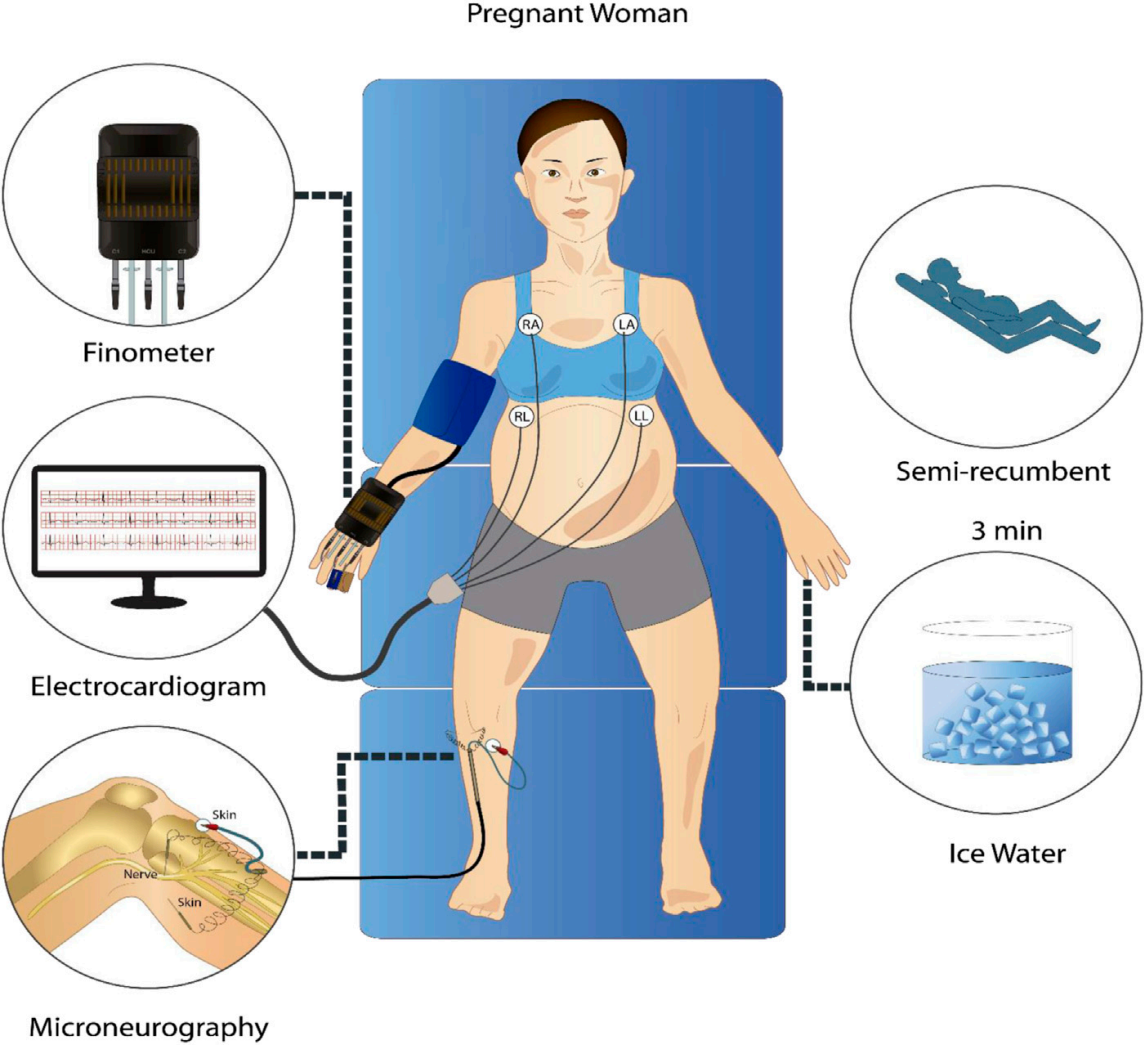
Representative schematic of the signal acquisition protocol. In a semi-recumbent position, participants underwent a 3-min rest period and a 3-min cold pressor test while electrocardiogram, continuous non-invasive blood pressure, and microneurography signals were recorded.

Subjects arrived at the laboratory at 8:00 a.m. after a 12-h fast. The consumption of caffeine or alcohol and the performance of strenuous exercise were not allowed during the previous 12 h. After a standardized breakfast and voiding their bladder, the participants rested semi-recumbent for the instrumentalization in a controlled environment with an ambient temperature of 20 °C and decreased ambient light and noise. For microneurography, the peroneal nerve was used to record MSNA at 10.000 Hz with a tungsten electrode with an uninsulated 1–5 μm tip at the fibular head and a reference electrode inserted 1–3 cm from the recording site (Mano et al., 2006). This technique allowed the acquisition of pulse-synchronous bursts of activity, characteristic of sympathetic vasoconstrictor neurons innervating smooth muscle (Vallbo et al., 2004). Finometer (Finapres Medical Systems, Amsterdam, The Netherlands) was used to measure continuous BP sampled at 1,000 Hz and was calibrated using three baseline BP values obtained using manual sphygmomanometry. Electrocardiogram (ECG) was measured using a standard 3-lead from the Finometer device at 1,000 Hz. The derived respiratory signal was obtained from the ECG Finometer signal with a sampling frequency of 1,000 Hz using the toolbox BioSigKit (Sedghamiz, 2018). The method used extracts morphological information from the whole QRS complexes using principal component analysis (PCA) and has shown good performance in terms of wave morphology similarities with direct measurements of the respiratory wave in controlled environments like ours (Varon et al., 2020). After instrumentation was complete, participants rested for a minimum of 10 min before baseline measures began. After 3 min of measurements at baseline, the cold pressor test (CPT) was performed, consisting of placing the left hand in ice water up to the wrist for 3 min. Figure 1 shows a schematic of the protocol and instrumentation used.

### 2.2 Data analysis

#### 2.2.1 Preprocessing

The MSNA signal was filtered with a low pass butterworth filter of 2000 Hz, rectified, and processed with a root mean square moving averaged window with a time constant of 200 ms, as described in the literature (Espinoza et al., 2019). The neurogram obtained was filtered with a band pass butterworth filter of 0.01–2 Hz. Then, the signal was resampled to 5 Hz. For the extraction of the RR interval, the ECG signal was filtered with a Butterworth bandpass between 0.5 Hz and 30 Hz; the maximal overlap discrete wavelet transform (MODWT) was used to enhance the R peaks in the ECG waveform (Zidelmal et al., 2012). Briefly, wavelet transform decomposes the signal into time-varying frequency (scale) components using a finite wave-like oscillation that can have different morphology (Stefanovska and Ci, 1998). The Wavelet transform separates signal components into different frequency bands, enabling a sparser signal representation. The wavelet selection should consider the feature’s morphology to be identified. Fourth-order Symlet Wavelet was chosen to enhance the R peaks in the ECG waveform because it resembles the QRS complex. The signal was reconstructed using only the coefficients on scales four and 5 (level of stretching or compressing of the wavelet) to maximize the energy of the QRS complex (Zidelmal et al., 2012). Then, a Matlab (The MathWorks Inc., Natick, United States) function based on the localization of local maxima (‘Findpeaks’) was used to identify the RR intervals. Once the RR interval signal was obtained, the Matlab function ‘Filloutliers’ was used to identify possible outliers in a moving window of 50 s. The outliers were defined as elements more than three scaled median absolute deviations from the median of the window and were removed and interpolated using Piecewise Cubic Hermite Interpolating Polynomial (PCHIP). We used a moving window of 50 s to look for these points because it gave a good performance capturing outliers without removing relevant signal behavior. Once the signal was clean, the resultant signal was interpolated–using retention of order 0–to obtain a sampling frequency of 5 Hz.

For the BP signal, systolic blood pressure (SBP) and diastolic blood pressure (DBP) values were obtained with a sampling rate of 1 Hz. Similar to the processing of the RR interval signal, the ‘Filloutliers’ function was used to identify, eliminate and interpolate outliers. The resultant SBP and DBP signals were interpolated with a PCHIP at 5 Hz. An ECG-derived respiratory signal was interpolated at 5 Hz and filtered with a passband between 0.04 and 0.4. Considering that all signals need to have the same sampling rate for the analysis, 5 Hz was chosen based on previous studies (Borovik et al., 2020).

#### 2.2.2 Synchronization

The synchronization phenomenon can be defined as the adjustment of oscillator rhythms due to some interaction between them (Pikovsky et al., 2001). This property has been studied in the field of neuroscience (Lowet et al., 2016) and the analysis of cardiorespiratory interaction. Phase synchronization implies that the oscillators have a specific relationship with each other and adjust their phases according to their differences (Pikovsky et al., 2001). This approach can be used to evaluate the dynamic relationship between cardiovascular, respiratory, and nervous components. We used continuous wavelet transform to perform a time-frequency analysis of the physiological signals. Wavelet coefficients were used to calculate the WPCO (Bandrivskyy et al., 2004), which allows for assessing the degree of phase synchronization between signals through the coherence between instantaneous phases of both variables. A constant difference between the phases implies high coherence between the signals that can be translated into high-phase synchronization (Cui et al., 2014). A brief description of the method is provided below.

#### 2.2.3 Wavelet transform

The wavelet transform can be used to analyze changes in signal frequency components over time (Cartas-Rosado et al., 2020) and consists of convoluting the signal under study with the complex conjugate of the family wavelet.

$$W_{\left(s,t\right)}=\frac{1}{\sqrt{s}}\int_{-\infty }^{+\infty }g\left(u\right)\Psi *\left(\frac{u-t}{s}\right)du$$ (1)

Where W (s, t) is a wavelet coefficient, and *Ψ* is the Morse mother wavelet, scaled by the factor s and translated in time by t.

In simple words, the wavelet is scaled (shrunk and expanded) to different levels with different frequency content and displaced along the time series. The result is a multiresolution analysis where each coefficient will have information about how much frequency information of the scaled wavelet is in the time series. Therefore, the coefficients W (s,t) have information about the amplitude and phase of each scale s at time t (Tankanag et al., 2020).

The synchrosqueezed wavelet transform (Daubechies et al., 2011) is an adaptation of the wavelet transform that reduces the scalogram’s redundant information and, thus, finds frequency bridges within the signal. The bridges represent dominant frequencies that could result from autonomic mechanisms such as sympathetic and parasympathetic control. Meignen et al. (Meignen et al., 2019) propose this approach to evaluate multifrequency signals that are an intrinsic characteristic of physiological signals. The synchrosqueezed transform was used here to inspect the signals in terms of their frequency behavior over time by observing the bands under study in the scalogram. The phase coherence calculations were performed using the continuous wavelet transform.

#### 2.2.4 Wavelet phase coherence

For each signal, the coefficients were obtained:

$$W_{\left(s_{k},t_{n}\right)}=a_{k,n}+{ib}_{k,n}$$ (2)

From which the instant phase was extracted as $\phi_{k,n}=\arctan\left(b_{k,n}/a_{k,n}\right)$ . Then, the relative phase was calculated as the difference between the instantaneous phases for each time and scale ${\Delta \phi }_{k,n}$ (Tankanag et al., 2014). Then,

$$\cos\left(\phi_{k,n}\right)=\frac{a_{1k,n}a_{2k,n}+b_{1k,n}b_{2k,n}}{\sqrt{a_{1k,n}^{2}+b_{1k,n}^{2}}\sqrt{a_{2k,n}^{2}+b_{2k,n}^{2}}}$$ (3.1)

$$\sin\left(\phi_{k,n}\right)=\frac{b_{1k,n}a_{2k,n}-a_{1k,n}b_{2k,n}}{\sqrt{a_{1k,n}^{2}+b_{1k,n}^{2}}\sqrt{a_{2k,n}^{2}+b_{2k,n}^{2}}}$$ (3.2)

the coefficients cos ${\Delta \phi }_{k,n}$ and sin ${\Delta \phi }_{k,n}$ were averaged over time for the length of the time series N (Tankanag et al., 2014):

$$\langle \cos{\Delta \phi }_{k,n}\rangle =\frac{1}{N}\overset{N}{\underset{n=1}{\sum }}\cos{\Delta \phi }_{k,n}$$ (4.1)

$$\langle \sin{\Delta \phi }_{k,n}\rangle =\frac{1}{N}\overset{N}{\underset{n=1}{\sum }}\sin{\Delta \phi }_{k,n}$$ (4.2)

Finally, the time-averaged wavelet phase coherence function is defined as

$$C_{\phi }\left(s_{k}\right)=\sqrt{{\langle \cos{\Delta \phi }_{k,n}\rangle }^{2}+{\langle \sin{\Delta \phi }_{k,n}\rangle }^{2}}$$ (5)

$C_{\phi }$ takes values from 0 to 1, where one indicates complete phase-locking and 0 indicates lack of phase synchronization between the signals for each frequency (Tan et al., 2016). With this approach, RR, SBP, DBP, MSNA, and respiratory signals were analyzed in a bivariate way.

#### 2.2.5 Surrogate data

When WPCO is evaluated, the null hypothesis is that there is no causal relationship among the signals (the phase coherence is not significantly different from zero). Also, because of the short length of the signals, high values of phase coherence can be observed in the low frequencies (Bandrivskyy et al., 2004). This occurs because low-frequency components are represented by fewer periods and, consequently, less variation of the phase is captured, which translates into an artificial increase of coherence. Surrogate data were generated using the Amplitude-adjusted Fourier transform (AAFT) approach (Bu et al., 2017) to help assess the statistical significance of the WPCO values obtained. One hundred surrogate values of WPCO were randomly generated, maintaining the same mean, variance, and autocorrelation function of the original sequence but modifying the phase, removing any phase relation (Schreiber and Schmitz, 2000). Original WPCO values were considered statistically significant when they were two standard deviations over the mean of the surrogate data (Tankanag et al., 2014). The median of the valid values of phase coherence in LF and HF bands was used as a representative marker for each range of frequencies to evaluate intra- and inter-group differences.

#### 2.2.6 Statistical analysis

Statistical data analysis was performed in Matlab (The MathWorks Inc., Natick, United States). Data did not show a normal distribution; therefore, non-parametric statistics were used. Median and 95% confidence intervals (CI) are presented. Kruskal–Wallis one-way analysis of variance was used to assess inter-group statistically significant differences (NP, HYP, and NNP) at rest and during CPT. Dunn-Sidak was used as a *post hoc* test to identify the groups with significant differences. On the other hand, Wilcoxon signed-rank test was used to reveal significant differences between baseline and CPT. Statistical significance was reached at *p* < 0.05.

## 3 Results

This study analyzed the phase coherence of cardiovascular, respiratory, and MSNA using the wavelet transform approach to elucidate differences in interaction and control mechanisms between NP, NNP, and HYP. Table 1 summarizes the sample’s demographic, anthropometric, and baseline characteristics. The NNP women were younger than the NP and HYP group, although all the participants were under 41 years old. Unsurprisingly, non-pregnant women weighted less than women in the pregnant groups. The NNP group shows significantly higher values of RR than the NP group (lower HR). As expected, the HYP group showed significantly higher values of SBP and DBP than NP and NNP. Regarding MSNA, the HYP group shows a higher but not statistically significant number of bursts per minute than the other two groups. No significant differences were found in respiratory rate.

**TABLE 1:** Participant anthropometrics and baseline characteristics. The table shows the results for normotensive non-pregnant women (NNP), normotensive pregnant women (NP), and hypertensive pregnant women (HYP) groups in terms of median and 95% CI. RR: RR interval. DBP: Diastolic blood pressure. SBP: Systolic blood pressure. MSNA: Muscle sympathetic nerve activity. RESP: Respiratory wave. *: significant difference with NP. †: significant difference with HYP. NA: not applicable.

	NNP	NP	HYP
Age	26† (23–28)	33 (28–34)	34 (29–37)
Height (cm)	165 (161–170)	173 (169–175)	164 (158–168)
Weight (kg)	63† (57–65)	85 (75–91)	93 (81–102)
Body mass index (Kg/m^2^)	22.6† (20.8–23.8)	27.3 (25.4–30.2)	34.1 (31.5–36.4)
Gestation (week)	NA	35 (34–36)	33 (31–36)
RR interval (ms)	890.07* (818.70–960.18)	711.7 (662.41–819.47)	718.15 (700.44–884.27)
DBP (mmHg)	69.52† (61.62–72.46)	69.49† (66.18–72.85)	84.98 (81.62–91.75)
SBP (mmHg)	106.69† (100.52–119.32)	104.97† (101.76–112.94)	135.13 (133.73–143.85)
MSNA (burst/min)	40.67 (31.00–54.00)	54.00 (40.67–65.33)	56.67 (31.00–68.67)
RESP (breaths/min)	11.33 (10.00–13.33)	11.17 (10.67–13.00)	10.00 (9.67–13.33)

**FIGURE 2:**
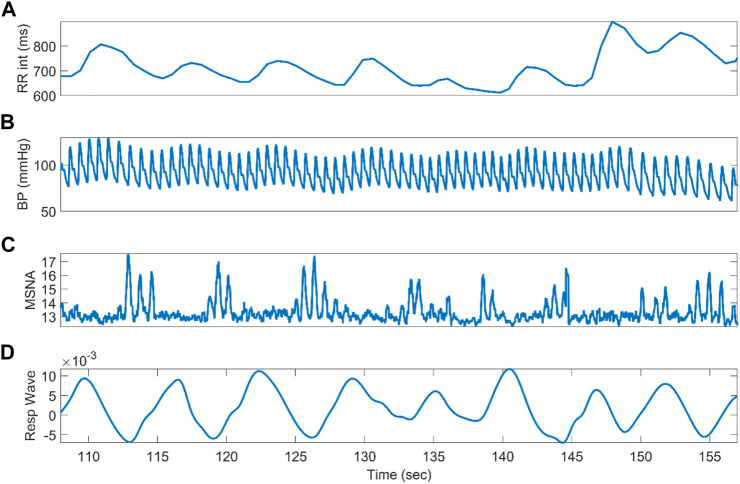
Physiological recordings from a representative normotensive pregnant woman at rest. From the top, it can be seen RR interval **(A)**, blood pressure wave **(B)**, integrated muscle sympathetic nerve activity **(C)**, and respiratory wave **(D)**.

Figure 2 depicts the RR interval, BP wave, integrated MSNA, and respiratory wave at rest for a representative NP subject. The signals show well-defined oscillatory patterns already described in the literature (Bu et al., 2017). The most obvious is the respiratory modulation of cardiac activity (Figure 2A), followed by the low-frequency oscillations in blood pressure (Figure 2B), which are likely related to baroreflex control. Figure 2C shows the neurogram from the MSNA signal. Depolarizations of the sympathetic fibers that result in bursts can be observed (Schreiber and Schmitz, 2000; Park et al., 2018; Dell’Oro et al., 2019; Laurin et al., 2017). Supplementary Figure S1 shows similar results for the NNP group in terms of BP. The HYP group also showed similar behavior but higher magnitudes for BP (Supplementary Figure S2).

**FIGURE 3:**
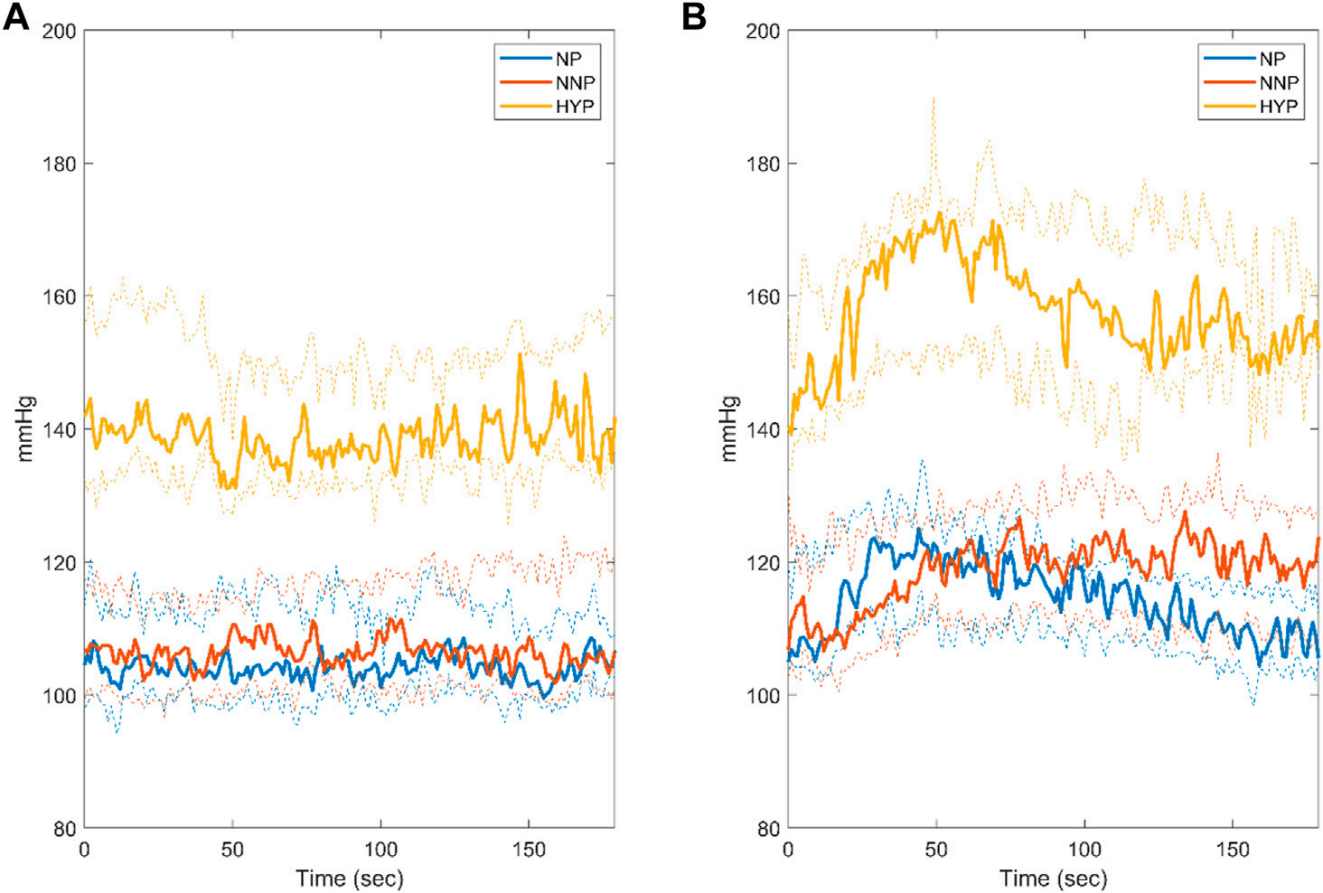
Systolic blood pressure response during rest **(A)** and cold pressor test **(B)** for normotensive pregnant women (NP, blue), normotensive non-pregnant women (NNP, red), and hypertensive pregnant women (HYP, yellow). The solid line shows the median with its 95% CI.

Figure 3 shows SBP dynamics for NP, NNP, and HYP groups at rest (Figure 3A) and during CPT (Figure 3B). The HYP group showed significantly higher median values than NP and NNP under both conditions. This was expected, given their pathology. In contrast, the NP and NNP groups did not show significant differences. During CPT, the HYP group showed a biphasic response to the stimulus (Usselman et al., 2015b), with a clear peak around 50 s with a 23.3% increase with respect to baseline and a slow recovery to 12.4% over the baseline in the last 20 s. On the other hand, the NP group peaks at around 50 s with a 19.3% increase from baseline, but a more efficient recovery, with a mean of 4.4% over the baseline in the last 20 s (See Supplementary Figure S3). NNP behaves similarly to HYP, but the peak was seen at around 70 s with an increase of 19.1%, maintaining SBP 11.9% over baseline at the last 20 s of the test. This value was significantly different from the NP group at the end of the test.

**FIGURE 4:**
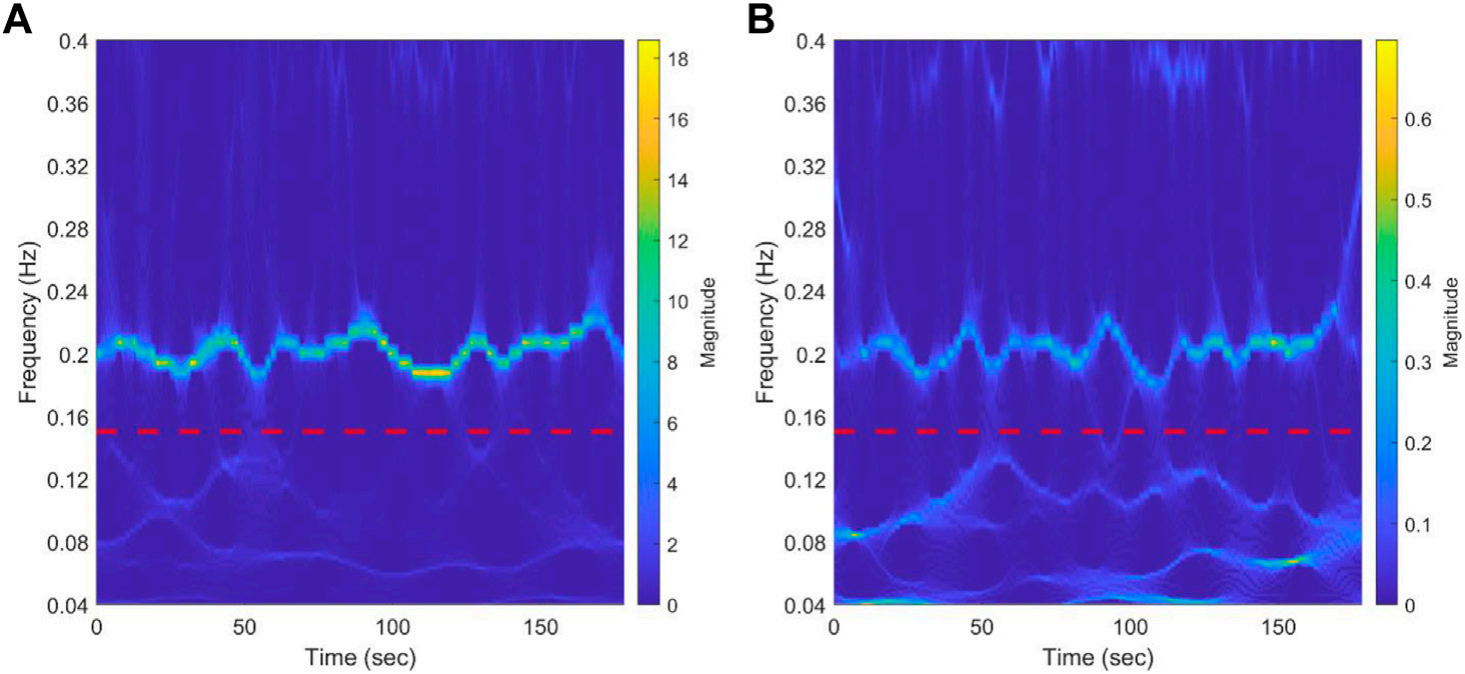
Results of synchrosqueezed transform of RR interval **(A)** and systolic blood pressure **(B)** during baseline from a representative normotensive pregnant woman. The red dotted line separates low-frequency bands (0.04-0.15 Hz) from the high-frequency band (0.15-0.4 Hz). A bridge representing respiration modulation in the HF band can be clearly seen. In the LF band, almost three bridges that cross each other can be identified.

Figure 4 shows the scalogram of RR during rest from a representative NP subject using synchrosqueezed wavelet transform. The red dotted line divides the spectrum in the HF and LF bands. A clear bridge can be seen in the HF, while almost three bridges crossing each other in the LF can be identified. This could be observed for most of the participants in the three groups (data not shown). The results for a representative NP subject during CPT can be seen in Supplementary Figure S4. The scalogram maintains the frequency bridges and the changes in the main frequency during the test in the HF band. The behavior in the LF is maintained with respect to baseline.

**FIGURE 5:**
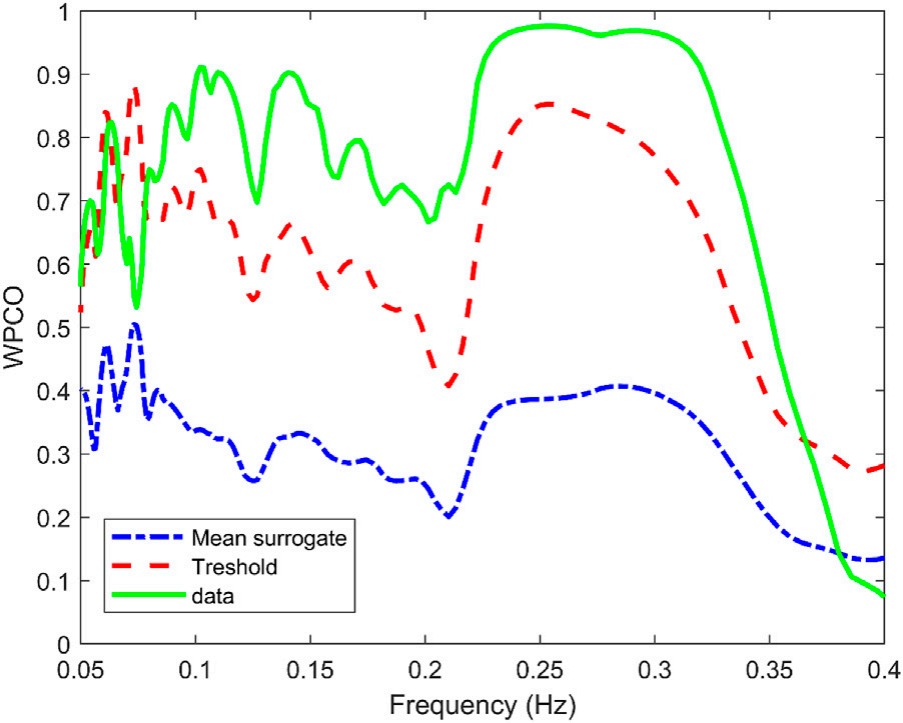
Wavelet phase coherence (WPCO) between the systolic blood pressure (SBP) and RR interval (solid green line) for a representative subject of the normotensive non-pregnant group. Moreover, WPCO between the SBP signals and the corresponding surrogates for the RR interval is shown. The mean (dash-dotted blue line) and mean±2σ (dashed red line) is given for the surrogates.

**FIGURE 6:**
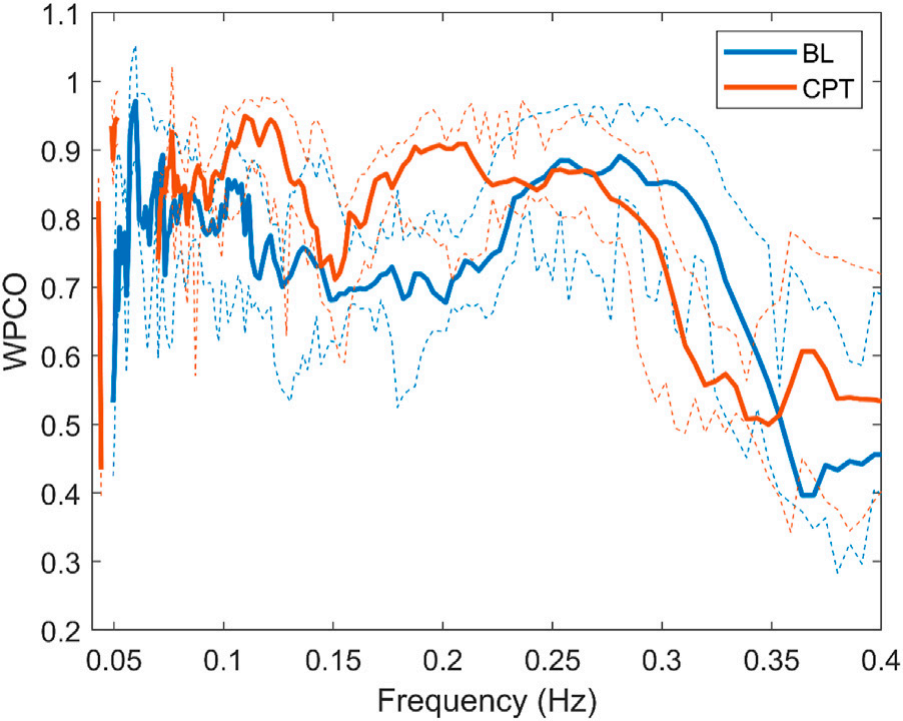
Wavelet phase coherence of RR interval and systolic blood pressure for the normotensive non-pregnant group during baseline (BL) and cold pressor test (CPT) in terms of median and 95% CI.

Surrogate data was used to verify that the correlation between the signals was statistically significant. Figure 5 shows the mean value of the surrogate data together with the limit defined to ensure significant values for a representative subject of the NNP group. It can be seen that WPCO values exceeded the limits of the surrogate data in the high and low-frequency bands. However, as the frequency decreases, the WPCO values cease to be significant. This might be due to the fact that the recording lasted only 3 min, which is too short to evaluate very low frequencies. The results for each group (median and 95% CI) at rest and during CPT in the HF and the LF bands are shown in Tables 2, 3, respectively. For illustrative purposes, Figure 6 shows the results of the WPCO between RR and SBP for the NNP group at rest and during CPT. Results for NP and HYP groups are shown in Supplementary Figures S5, 6, respectively. Differences in coherence can be observed throughout the spectrum.

**TABLE 2:** Wavelet phase coherence results in the high-frequency band. The table shows the results for normotensive non-pregnant women (NNP), normotensive pregnant women (NP), and hypertensive pregnant women (HYP) during baseline (BL) and cold pressor test (CPT) in terms of median and 95% CI. RR: RR interval. DBP: Diastolic blood pressure. SBP: Systolic blood pressure. MSNA: Muscle sympathetic nerve activity. RESP: Respiratory wave. *: significant difference with CPT. †: significant difference with NNP. λ: Significant difference with HYP.

		RR-SBP	RR-DBP	RR-MSNA	RR-RESP	SBP-MSNA	SBP-RESP	DBP-MSNA	DBP-RESP	MSNA-RESP
NP	**BL**	0.76 (0.68–0.87)	0.77 (0.66–0.86)	0.74† (0.61–0.85)	0.77 (0.60–0.91)	0.77† (0.62–0.84)	0.73 (0.63–0.88)	0.70† (0.66–0.83)	0.73 (0.64–0.86)	0.72† (0.62–0.82)
	**CPT**	0.78 (0.62–0.85)	0.82 (0.66–0.83)	0.72 (0.70–0.79)	0.81 (0.62–0.84)	0.78 (0.54–0.82)	0.83 (0.70–0.86)	0.77 (0.62–0.81)	0.82 (0.51–0.84)	0.71 (0.59–0.83)
NNP	**BL**	0.75* (0.68–0.78)	0.73 (0.63–0.77)	0.57* (0.46–0.63)	0.63 (0.56–0.76)	0.61* (0.50–0.66)	0.68 (0.52–0.78)	0.60* (0.50–0.62)	0.58 (0.49–0.71)	0.48* (0.45–0.58)
	**CPT**	0.83 (0.75–0.89)	0.81 (0.70–0.86)	0.71^λ^ (0.63–0.76)	0.71 (0.67–0.87)	0.68^λ^ (0.64–0.72)	0.73 (0.66–0.88)	0.66^λ^ (0.63–0.72)	0.73 (0.53–0.87)	0.67 (0.60–0.77)
HYP	**BL**	0.72 (0.66–0.77)	0.78 (0.67–0.79)	0.61 (0.55–0.70)	0.59 (0.57–0.77)	0.62 (0.54–0.76)	0.70 (0.57–0.83)	0.69 (0.54–0.74)	0.61 (0.49–0.77)	0.52 (0.38–0.74)
	**CPT**	0.67 (0.63–0.85)	0.75 (0.58–0.82)	0.56 (0.42–0.69)	0.66 (0.54–0.76)	0.50 (0.41–0.70)	0.63 (0.56–0.81)	0.55 (0.49–0.67)	0.58 (0.50–0.77)	0.62 (0.54–0.67)

**TABLE 3:** Wavelet phase coherence results in the low-frequency band. The table shows the results for normotensive non-pregnant women (NNP), normotensive pregnant women (NP), and hypertensive pregnant women (HYP) during baseline (BL) and cold pressor test (CPT) in terms of median and 95% CI. RR: RR interval. DBP: Diastolic blood pressure. SBP: Systolic blood pressure. MSNA: Muscle sympathetic nerve activity. RESP: Respiratory wave. *: significant difference with CPT. †: significant difference with NNP.

		RR-SBP	RR-DBP	RR-MSNA	RR-RESP	SBP-MSNA	SBP-RESP	DBP-MSNA	DBP-RESP	MSNA-RESP
NP	**BL**	0.68 (0.59–0.81)	0.71* (0.60–0.76)	0.68 (0.58–0.73)	0.77 (0.61–0.81)	0.68 (0.64–0.86)	0.68 (0.58–0.77)	0.76 (0.72–0.81)	0.71† (0.59–0.77)	0.65 (0.47–0.72)
	**CPT**	0.74 (0.71–0.86)	0.78 (0.76–0.84)	0.76 (0.66–0.87)	0.73 (0.67–0.80)	0.76 (0.68–0.85)	0.70 (0.63–0.77)	0.79 (0.74–0.82)	0.73 (0.70–0.77)	0.61 (0.59–0.78)
NNP	**BL**	0.73 (0.62–0.82)	0.76 (0.66–0.81)	0.74 (0.54–0.83)	0.69 (0.57–0.72)	0.78 (0.66–0.87)	0.70 (0.53–0.83)	0.76 (0.62–0.78)	0.58 (0.54–0.68)	0.64 (0.56–0.76)
	**CPT**	0.82 (0.75–0.84)	0.81 (0.69–0.88)	0.77 (0.68–0.89)	0.80 (0.64–0.86)	0.78 (0.69–0.86)	0.76 (0.56–0.88)	0.80 (0.75–0.89)	0.68 (0.60–0.76)	0.72 (0.65–0.98)
HYP	**BL**	0.65 (0.43–0.79)	0.66 (0.52–0.88)	0.62 (0.51–0.74)	0.61 (0.60–0.62)	0.56 (0.53–0.72)	0.00 (0.00–0.00)	0.63 (0.47–0.69)	0.00 (0.00–0.00)	0.00 (0.00–0.00)
	**CPT**	0.76 (0.63–0.88)	0.76 (0.60–0.94)	0.69 (0.62–0.75)	0.86 (0.77–0.95)	0.65 (0.55–0.82)	0.73 (0.62–0.93)	0.69 (0.67–0.80)	0.80 (0.70–0.90)	0.70 (0.56–0.84)

**FIGURE 7:**
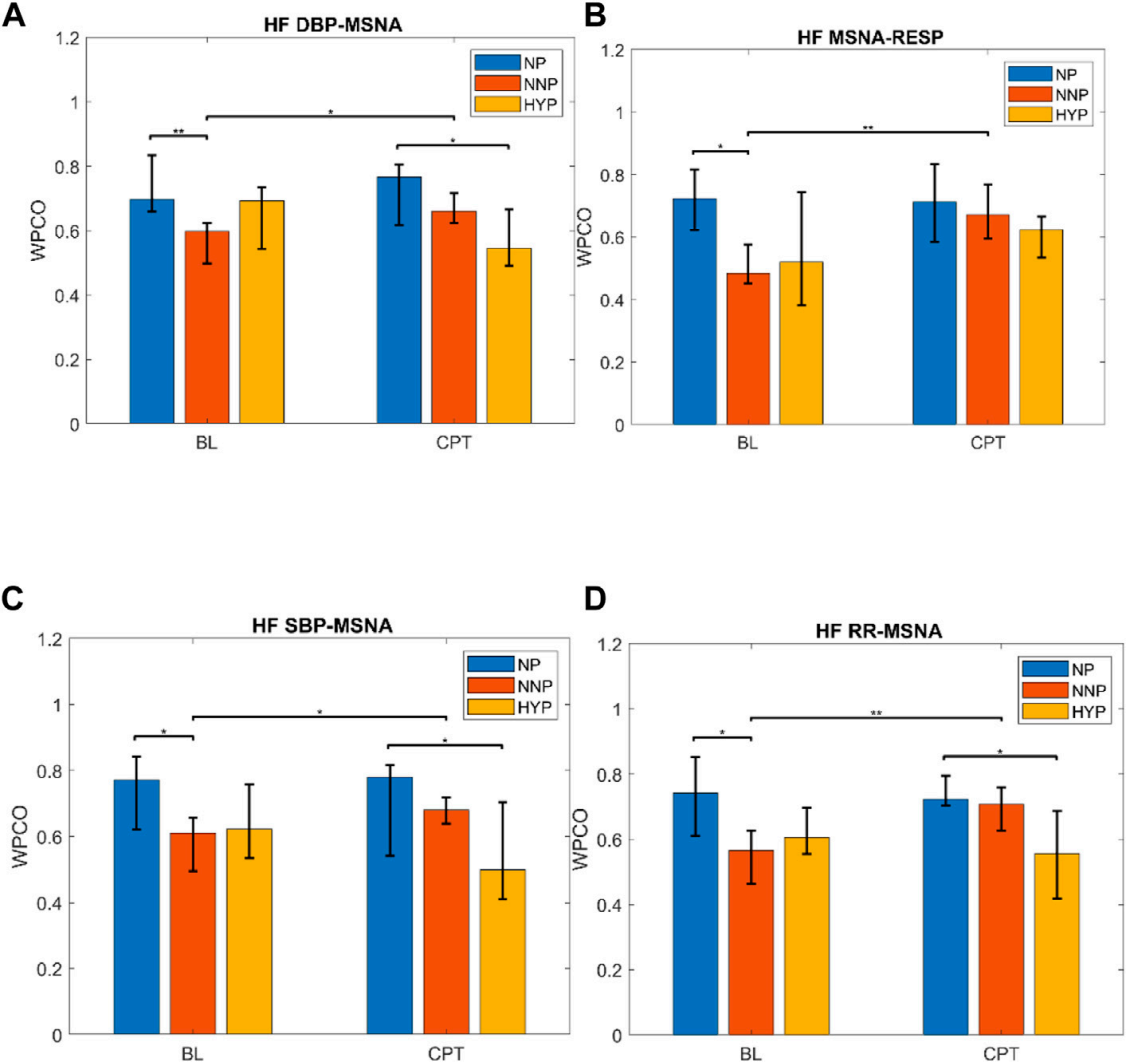
Wavelet phase coherence (WPCO) results for normotensive pregnant women (NP), normotensive non-pregnant women (NNP), and hypertensive pregnant women (HYP) in terms of median and 95% CI during baseline (BL) and cold pressor test (CPT). Results show WPCO between muscle sympathetic nerve activity (MSNA) and diastolic blood pressure **(A)**, MSNA and respiratory wave (RESP) **(B)**, MSNA and systolic blood pressure SBP **(C)**, and MSNA and RR interval **(D)** in the high-frequency band (HF). * Represents *p* < 0.05. ** represents *p* < 0.01.

The most relevant findings are summarized in Figure 7. Significant differences are observed in the HF band in MSNA-DBP phase coherence between NP and NNP at rest (NP 0.70 (0.66–0.83) vs NNP 0.60 (0.50–0.62) and between NP and HYP during CPT (NP 0.77 (0.62–0.81) vs HYP 0.55 (0.49–0.67) (Figure 7A). NP showed significantly higher phase coherence between respiration and MSNA compared with NNP at rest (NP 0.72 (0.62–0.82) vs NNP 0.48 (0.45–0.58)) (Figure 7B). Figure 7C shows a significant increase in phase coherence between MSNA and SBP for the NNP group during the CPT, while NP tends to maintain some stability in the WPCO values. HYP shows a slight non-significant drop. Finally, regarding RR-MSNA (Figure 7D), NP shows significantly higher values of phase coherence, but just with respect to NNP at rest (NP 0.74 (0.61–0.85) vs NNP 0.57 (0.46–0.63)), which reflects the increased sympathetic activity that affect cardiac baroreflex and vasomotor control during pregnancy. During CPT, NP and NNP show similar behavior, and HYP remains significantly lower compared with NP group (NP 0.72 (0.70–0.79) vs HYP 0.56 (0.42–0.69)). Moreover, only the NNP group shows a significant increase in WPCO after CPT.

In the LF band, the NP group showed significantly lower RR-DBP synchronization at rest than during CPT (rest 0.71 (0.60–0.76) vs CPT 0.78 (0.76–0.84)) (see Supplementary Figure S7). Moreover, normotensive pregnant women showed significantly higher RESP-DBP synchronization than the normotensive non-pregnant group at rest (NP 0.71 (0.59–0.77) vs NNP 0.58 (0.54–0.68)). Not enough HYP data were found to compare with the other two groups (see Supplementary Figure S8; Supplementary Table S1).

For additional information, Supplementary Tables S2, 3 summarize the percentage of outliers of the phase coherence result for each band, and Supplementary Tables S4, 5; Supplementary Figure S9 show the results in terms of median and interquartile range.

## 4 Discussion

The study of the pathophysiological mechanisms behind arterial hypertension in pregnant women and the physiological changes that pregnancy entails have been widely discussed over the years. While most of these studies focus on analyzing cardiovascular and nervous variables in terms of the magnitude of the effect of some stressors (Park et al., 2018; Dell’Oro et al., 2019; Laurin et al., 2017; Wehrwein and Joyner, 2013; Marchi et al., 2015; Béchir et al., 2003), the present study focused on studying the dynamics of the interactions between the cardiovascular, respiratory, and nervous systems through wavelet phase coherence analysis. The results showed that in the HF band: i) normotensive pregnant women have a higher coupling between MSNA and respiration at rest than normotensive non-pregnant women; ii) normotensive pregnant women have a higher coupling of MSNA-RR and MSNA-BP than normotensive non-pregnant women at rest; iii) hypertensive pregnant women have a lower coupling of MSNA-RR and MSNA-BP than normotensive pregnant women during CPT. The respiratory and sympathetic coupling increase in normotensive pregnant women reflects a stronger interaction between respiration and sympathetic output that could originate at the brainstem level. Changes in this interaction suggest physiological adjustments not previously described that could be a marker of a correct adaptation to pregnancy. On the other hand, the lower coupling between RR-MSNA and BP-MSNA in hypertensive pregnant women during the cardiovascular stressor reveals that the respiratory modulation of the nervous and cardiovascular systems is blunted with respect to normotensive pregnant women. This loss in the phase interaction can be interpreted as a mismatch in cardiovagal modulation and respiratory-sympathetic nerve dynamics. This is the first study to describe these differences that contribute to finding new biomarkers for early diagnosis of preeclampsia.

### 4.1 Cold pressor test response

The response to CPT of the three groups studied was consistent with what was stated by other authors. The biphasic response (Figure 3B), described by Usselman et al. (Usselman et al., 2015b), shows the effects of sympathetic activation and its subsequent regulation by baroreflex. The HYP group showed the greatest increase in SBP values, remaining over 10% from baseline at the end of the test. On the other hand, NP showed a blunted increase in SBP that decreased to 4.1% over the baseline at the end of the test. Woisetschläger et al. (Woisetschläger et al., 2000) reported that pregnant women with preeclampsia showed greater vascular reactivity prior to developing symptoms, which suggests its use as a predictive tool for disease development. In the same way, Prachi et al. (Shelke et al., 2014) observed an elevated response to CPT in women with preeclampsia. Steinback et al. (Usselman et al., 2015a) evaluated the neurovascular transduction during CPT in normotensive pregnant and normotensive non-pregnant women observing a decrease in nerve signal transduction that translated into a blunted increase in BP in the first group. This evidence shows that CPT could be a suitable clinical test for the early detection and control of hypertensive disorder during pregnancy. Our results are consistent with these findings, so evaluating the interaction of the respiratory systems, nervous and cardiovascular during this test can give valuable information about the regulatory mechanisms involved.

### 4.2 Time-frequency analysis

To carry out the analysis in terms of phase, it is necessary to extract its value accurately. However, the method selected must consider the nature of the signals under study to avoid misleading conclusions. The present study used the synchrosqueezed wavelet transform (Daubechies et al., 2011; Meignen et al., 2019), a time-frequency analysis tool, to inspect the characteristics of the signals recorded in each participant and define the best approach to use. From this analysis, we observed that: i) the signals obtained do not have only one dominant frequency; multiple frequency components can be found at the same frequency band (LF and HF bands); ii) the frequency bridges cross each other, which makes it very difficult to follow their trajectories to estimate the instantaneous phases; iii) averaging the phases for each time column of the scalogram does not provide a reliable estimate of a representative phase of the studied frequency band. Inspecting the signals using this technique allowed us to select a method capable of comparing the phase locking of all the spectrum of frequencies of interest to obtain a more representative marker of signal interaction.

### 4.3 Wavelet phase coherence results at rest

The phase synchronization approach has been widely used to study the interdependence of cardiovascular signals in subjects with syncope (Ocon et al., 2009), microvasculature control (Tankanag et al., 2020), neuroscience (Mezeiová and Paluš, 2012), heart failure (Kuhnhold et al., 2017), and pregnancy (Moertl et al., 2013). Considering the multifrequency character of the cardiovascular signals, WPCO is a suitable tool to evaluate phase locking and, therefore, a phase synchronization marker (Cui et al., 2014). This approach has the advantage over spectral coherence that it finds locally phase-locked behavior (Cui et al., 2014). Spectral coherence allows evaluating the coherence of the signals in terms of frequency but does not provide phase information; therefore, it cannot be determined if the systems are coupled, which is a requirement for the study of synchronization (Rosenblum et al., 2001).

**FIGURE 8:**
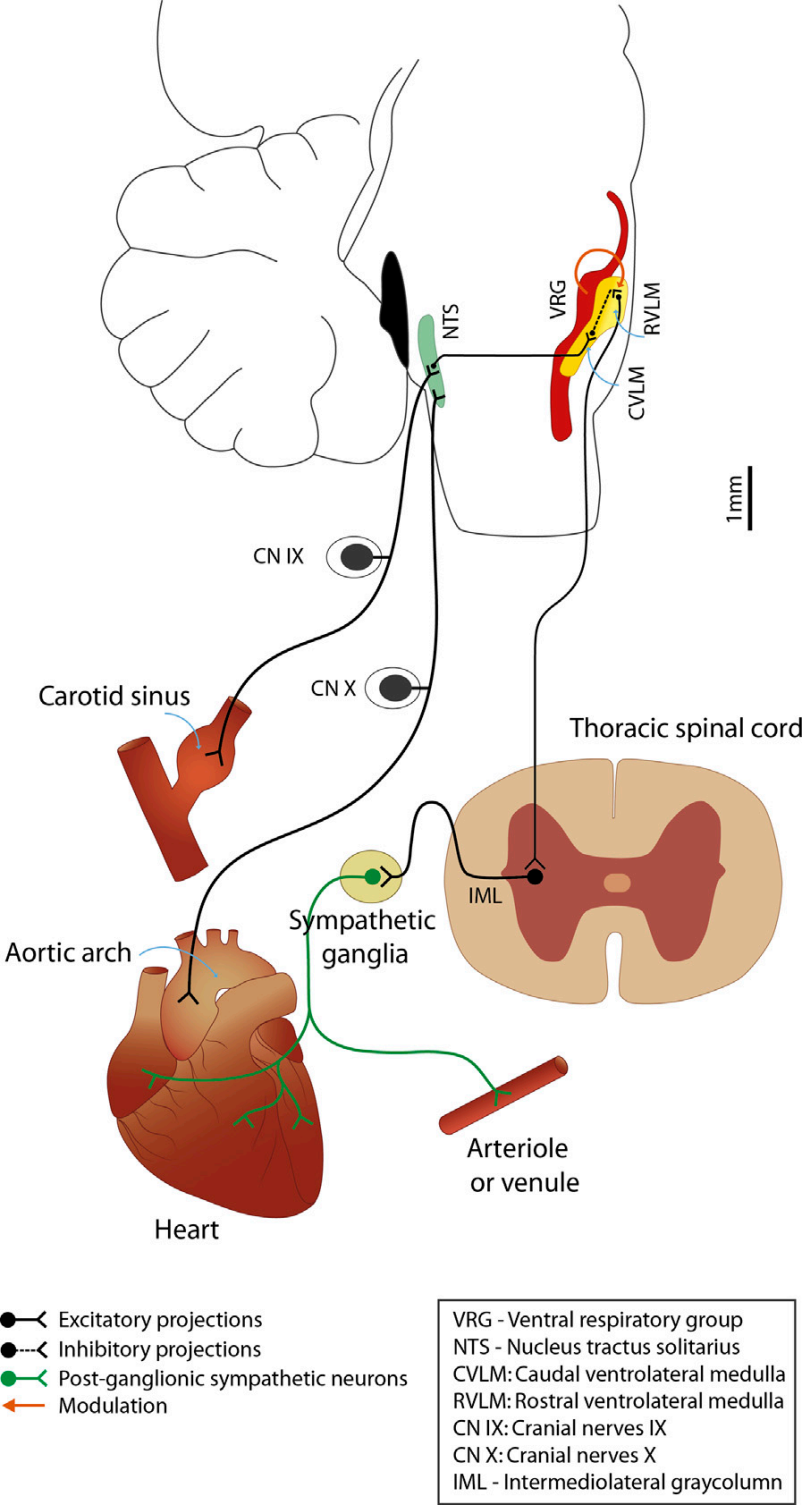
A schematic representation of the autonomic nervous system regulation of the cardiovascular system. Changes in blood pressure produce changes in the diameter of the carotid arteries and aorta, sensed by mechanoreceptors in the carotid sinus and aortic arch. This information travels through the afferent pathways: vagus nerve (CNX) and glossopharyngeal nerve (CNIX), to the main integrating center of cardiovascular control: the nucleus of the solitary tract (NTS). The NTS regulates the activity of the rostral ventrolateral medulla (RVLM) through inhibitory neurons in the caudal ventrolateral medulla (CVLM). RVLM is the main regulatory center of sympathetic output, and its activity modulates vascular tone and cardiac activity through efferent pathways. Close to RVLM is the ventral respiratory group (VRG), a column of neurons that fire action potentials in phase with respiration and which, due to its proximity to RVLM, could be responsible for the modulation of sympathetic output during respiration.

The WPCO results revealed increased respiratory modulation of sympathetic output in NP compared to NNP. This cardiovascular interaction has been extensively studied in animal and human models. While in anesthetized mice, an increase in MSNA was found during inspiration and in synchrony with phrenic nerve activity, in humans, the relationship between the signals is out of phase, finding the nadir of MSNA during inspiration and the sympathetic output peak at the end of expiration (Dempsey et al., 2002). The mechanisms behind this modulation are not yet clear; some authors suggest a baroreceptor interaction with lung stretch receptors, chemoreceptor modulation, and central interaction. Spradley et al. (Spradley, 2019) showed that baroreflex sensitivity in healthy pregnant women was not altered, while Garg et al. (Garg et al., 2020) reported a decrease in cardiovagal modulation and baroreflex sensitivity towards the end of a healthy pregnancy. Corrêa et al. (Da Silva Corrêa et al., 2019) found a decrease in parasympathetic modulation and baroreflex sensitivity of healthy pregnancy at rest but a preserved baroreflex response during posture changes. Usselman et al. (Usselman et al., 2015b) found maintenance of cardiovagal baroreflex but decreased sympathetic baroreflex gain, indicating that the baroreflex might not be the main mechanism of MSNA modulation. Results are controversial because baroreflex sensitivity has reliability and reproducibility problems, and cross-correlation methods do not quantify the causal relationship between BP and HR variability. On the other hand, functional changes in the controlling centers related to sympathetic output and respiration could be responsible for this sympathetic modulation (Zoccal et al., 2014). Previous work (Moraes et al., 2013) supports the idea that, due to the proximity of the ventrolateral medulla (RVLM) and the ventral respiratory group (VRG), projections from VRG to RVLM are the main responsible for this interaction (Figure 8). Our results show an increased relationship between the respiratory and nervous systems during a healthy pregnancy. The origin of this relationship could be a combination of both modulatory feedback signals from peripheral reflexes and central interaction. More research is necessary to understand how this relationship changes.

Results in NP at rest show a strong MSNA-RR phase coherence. Previous evidence of this relationship was reported by (Pagani et al., 1997), who observed that an increase or decrease in MSNA affected LF and HF power of RR, BP, and MSNA. Also, they showed that MSNA effectively represents the overall sympathetic activity in the body and suggests possible common central mechanisms for parasympathetic and sympathetic branches. However, these methods do not provide causality information on the variability of RR and MSNA, and the effect of one over the other cannot be quantified (Clemson et al., 2022). Our results overcome this disadvantage using a non-linear approach and show the autonomic cardiac modulation coupled to MSNA activity in the HF band. From this, we can conclude an increased cardiorespiratory coupling in normotensive pregnant women at rest according to the following: i) The increase of the sympathetic and respiratory coupling should lead to an increase of the high frequencies of MSNA according to the theory of self-sustained oscillators interaction (Rosenblum et al., 2001) ii) The increase of the sympathetic and cardiac coupling implies an increase of the vagal response for increase the energy of oscillatory cardiac components in the HF band. These results contrast with what was found in a previous study (Garg et al., 2020), where a decrease in cardiovagal modulation was observed along with a decreased BRS. However, as mentioned above, baroreflex sensitivity has reliability and reproducibility issues, and cross-correlation methods do not quantify the causal relationship between BP and HR variability. Finally, increased BP-MSNA coupling in NP at rest can be related to the effect of MSNA respiratory modulation by its effect on vasoconstriction.

### 4.4 Wavelet phase coherence results during CPT

During CPT, the HYP group suffered a decrease in phase coherence between RR-MSNA and BP-MSNA in the HF band. This test allows evaluation of the sympathetic output and the ability to regulate the blood pressure rise. As previously mentioned, our data show that during a stressor such as CPT, the cardiorespiratory coupling of HYP is weaker than NP. Chaswal et al. (Chaswal et al., 2018) reported that pregnancy reduces cardiovagal modulation, which becomes exacerbated in women with preeclampsia. Our results provide a new perspective about this dynamic, showing that for HYP the effect of respiration over cardiovascular control is blunted (see Figure 6). In the same way, the fall in WPCO for MSNA-BP in the HYP group reinforces the hypothesis of impairment in cardiovascular modulation by the nervous system. The response of the HYP group suggests that the maladjustment of autonomic control could be reflected in changes in the dynamic between respiration, nervous and cardiovascular systems.

The significant differences found in the LF band showed a slight but significant increase in the WPCO of NP compared to NNP in RESP-DBP. The synchronization of low frequencies with respiration is possible (Reyes et al., 2018), but the low number of subjects with significant values in the LF band (Supplementary Table S1) makes interpretation difficult. The short time recording can lead to overestimations of coherence due to the low number of cycles studied and the loss of significant measurements of WPCO. Future studies should consider broader time windows to analyze this frequency band better.

## 5 Limitations

Our study has several limitations. First, ECG-Derived Respiration was used as an approximation of the respiratory wave. To maximize the reliability of the extracted instantaneous respiratory phase, we used a method that extracts morphological information from the whole QRS complexes using principal component analysis (PCA) using the toolbox BioSigKit (Sedghamiz, 2018). Since this method is not based on R-R fluctuations but on the global variation of the amplitude of the QRS complex caused by impedance changes due to variations in lung volumes, it is not affected by autonomic control (Stefanovska and Ci, 1998). In a recent study, Varon et al. (Stefanovska and Ci, 1998) compared the performance of ten different techniques for deriving respiration from the electrocardiogram in multiple conditions, including different recording systems and breathing patterns. The results showed that the method we used (PCA-based) was among the best in terms of similarity and signal quality regarding respiratory waves. The PCA-based method behaves well for normal respiratory patterns and with clean signals since it is sensitive to outliers. However, in our experimental protocol, the measurements were made in a controlled environment and required avoiding any movement of the participant due to the complexity of the microneurography technique. Moreover, we performed manual correction of outliers from the electrocardiogram recordings. Finally, the evidence shows that, for estimating phases, the PCA method provides reliable values in terms of wave morphology, for which its use in this study is justified. Previous studies have also used ECG-Derived Respiration to study synchronization, showing results consistent with previous research (Moertl et al., 2013; Kuhnhold et al., 2017).

The second limitation is the limited recording time (3 min for baseline and CPT) which allows getting novel information about the interaction in the HF band due to the number of cycles under study but was too short to obtain more information on the LF band. Third, using CPT gives relevant information but does not allow isolating the response from all the other reflexes acting during the test. However, the use of more invasive tools or vasoactive drugs implies a risk for the population sample, given its state of vulnerability. Therefore, this simple but well-known test offers a valuable tool.

## 6 Conclusion

This is the first study to analyze the dynamics of the physiological oscillations in the cardiovascular, nervous, and respiratory systems in normotensive pregnant women, normotensive non-pregnant women, and hypertensive pregnant women. A direct measure of the activity of the peripheral sympathetic system was incorporated. The study was based on the use of complex signal analysis techniques that allowed the extraction of information not evident to the naked eye to unravel changes in cardiovascular control mechanisms. The results showed an increase in phase coherence between MSNA and RR interval signals, BP, and respiration, in the high-frequency band (0.15-0.4 Hz) in normotensive pregnant women compared to normotensive non-pregnant women during rest and compared to hypertensive pregnant women during the cold pressor test. These phase coherence values reveal increased cardiac parasympathetic modulation and higher respiratory-driven modulation of sympathetic vascular control in normotensive pregnant women, which could reflect their effectiveness in maintaining adequate blood pressure levels. A loss in this phase interaction could be interpreted as a maladjustment in cardiovagal modulation and respiratory-sympathetic nerve dynamics that characterize pregnant women with hypertensive disorders. Moreover, increased MSNA respiratory-driven modulation may give evidence of adaptative changes in the central nervous system to face pregnancy. More detailed studies are needed to determine the causal influences of cardiorespiratory coupling during pregnancy and its usefulness in the early diagnosis of preeclampsia.

## Data Availability

The raw data supporting the conclusion of this article will be made available by the authors, without undue reservation.
