# Supplementary material for: Increased respiratory modulation of cardiovascular control reflects improved blood pressure regulation in pregnancy
Source: Front Physiol. 2023 Mar 21;14:1070368. doi: 10.3389/fphys.2023.1070368 (PMC10070987; doi:10.3389/fphys.2023.1070368)
Supplement: Supplementary file 1 [file DataSheet1.PDF]

Increased respiratory modulation of cardiovascular control reflects improved blood pressure regulation in pregnancy

Martín Miranda Hurtado, Craig D. Steinback, Margie H. Davenport, Maria Rodriguez-Fernandez

Supplementary Material

1 Supplementary Figures and Tables

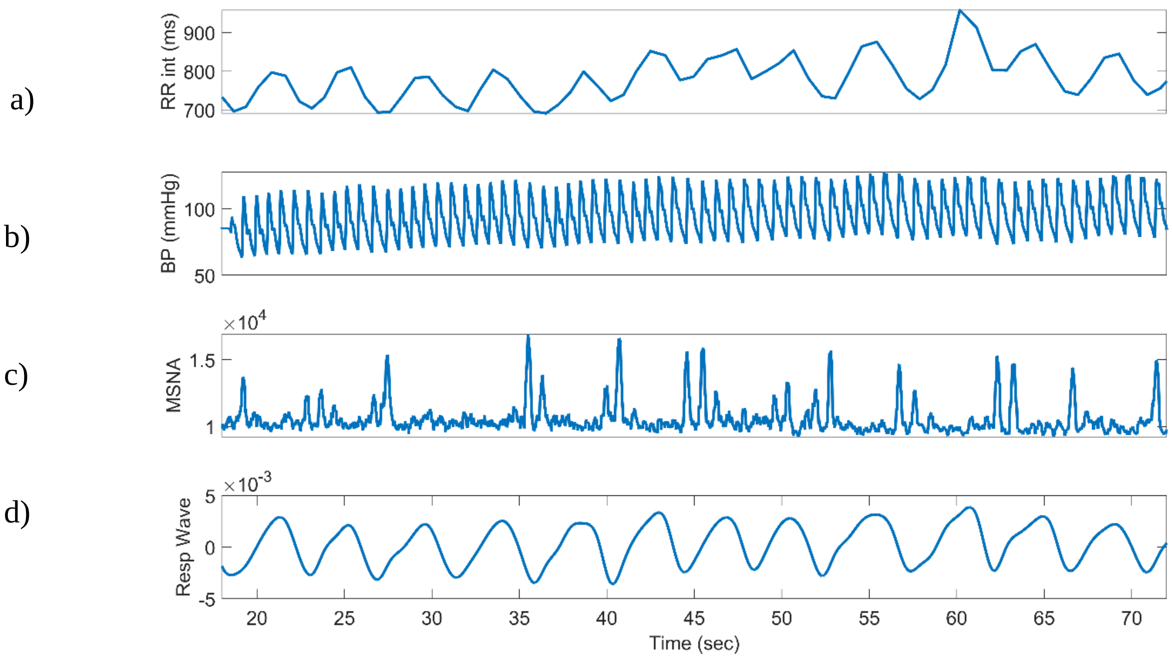

Figure S1. Physiological recordings from a representative normotensive non-pregnant woman at rest. From the top, it can be seen RR interval (a), blood pressure wave (b), integrated muscle sympathetic nerve activity (c), and respiratory wave (d).

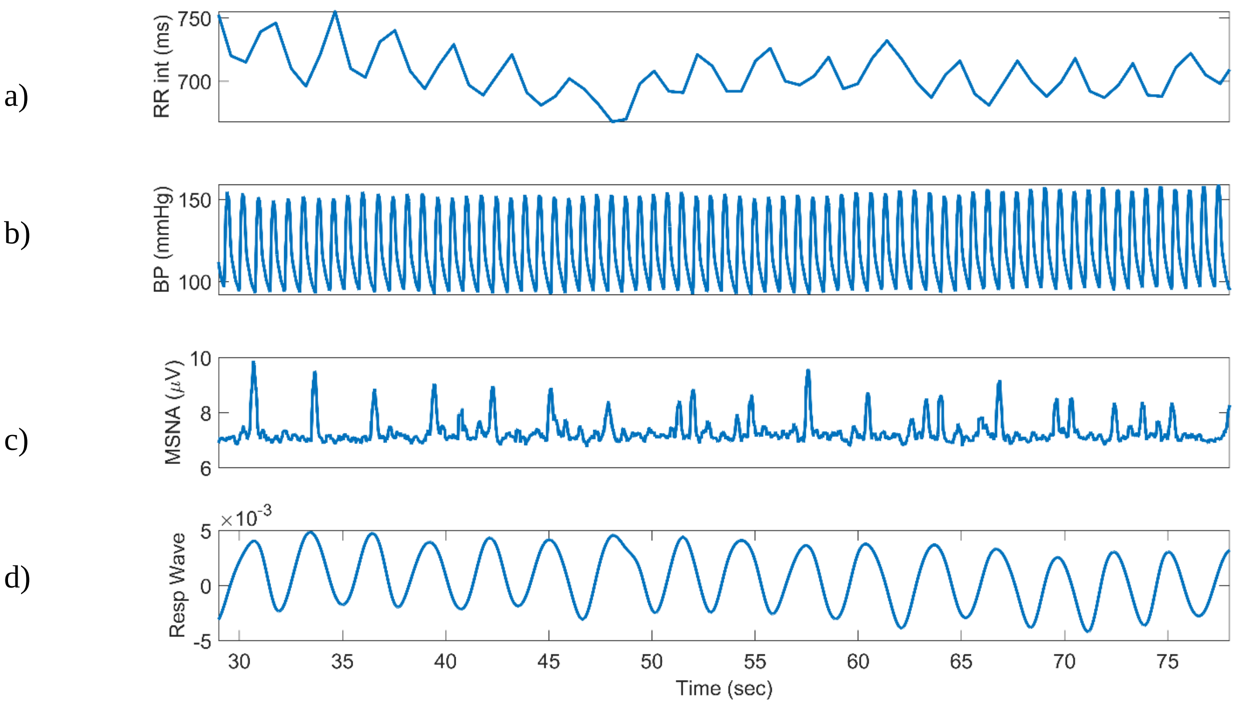

Figure S2. Physiological recordings from a representative hypertensive pregnant woman at rest. From the top, it can be seen RR interval (a), blood pressure wave (b), integrated muscle sympathetic nerve activity (c), and respiratory wave (d).

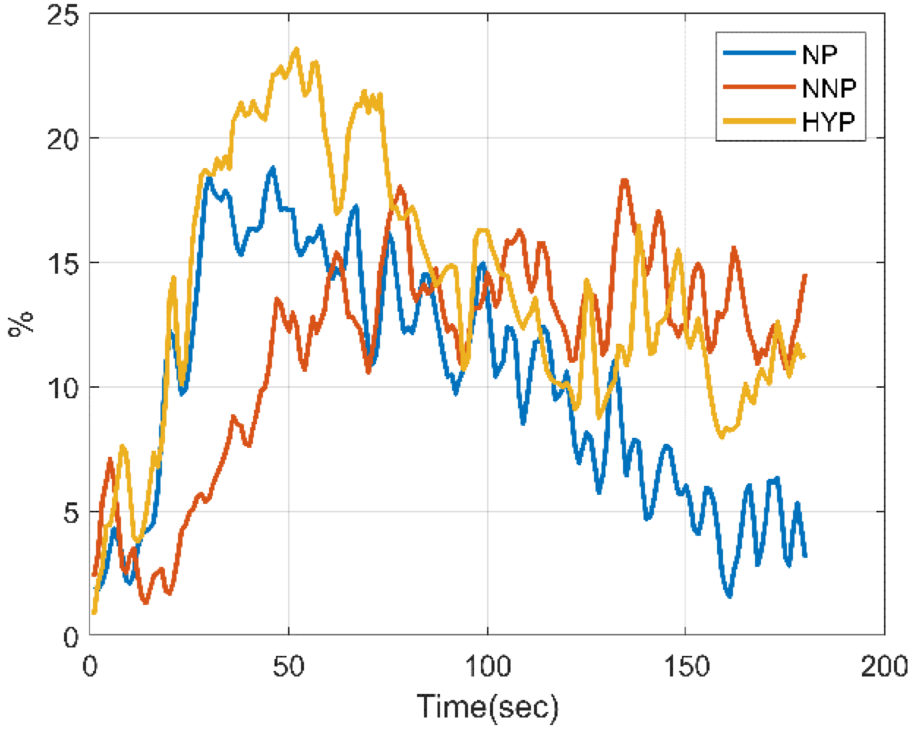

Figure S3. Response to cold pressor in terms of percentage increase from baseline in systolic blood pressure. Curves with a mean moving window of 3 seconds or normotensive pregnant women (NP), normotensive non-pregnant women (NNP), and hypertensive pregnant women (HYP).

a) b)

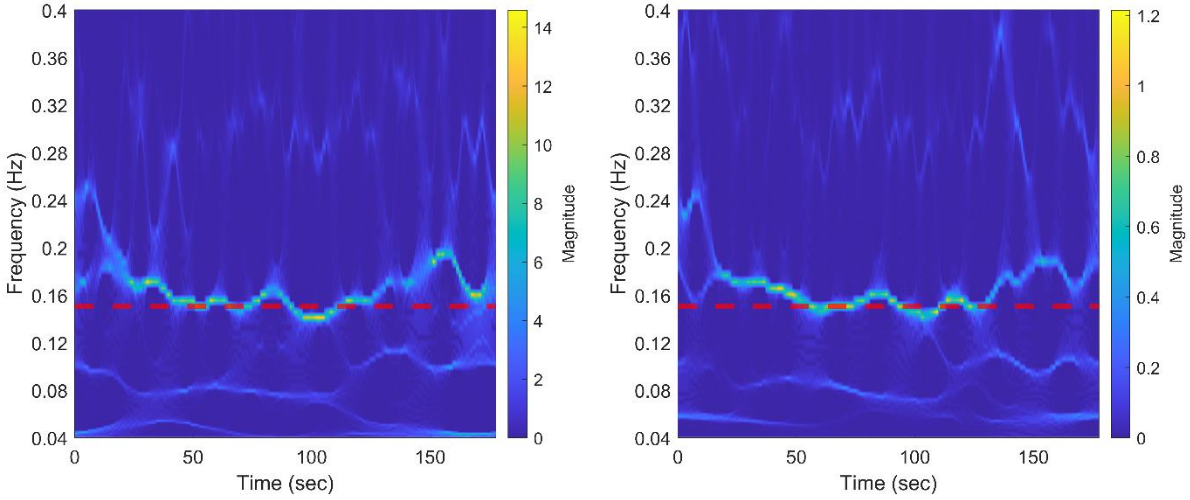

Figure S4. Results of synchrosqueezed transform from a representative normotensive pregnant subject during cold pressor test. (a) RR interval; (b) systolic blood pressure. The red dotted line separates the low-frequency band (0.04-0.15 Hz) from the high-frequency band (0.15-0.4 Hz). A bridge representing respiration modulation can be clearly seen in the HF band. In the LF band, almost three bridges that cross each other can be identified.

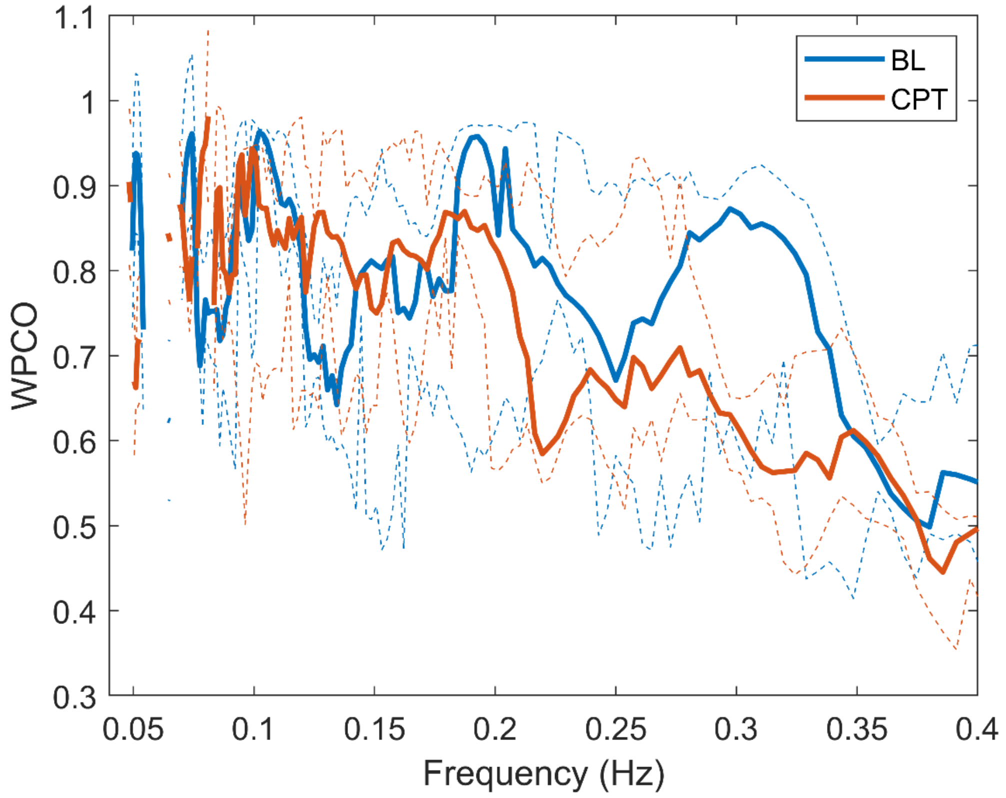

Figure S5. Wavelet phase coherence of RR interval and systolic blood pressure for the normotensive pregnant group during baseline (BL) and cold pressor test (CPT) in terms of median and 95% IC. *Indicates significant differences between BL and CPT.

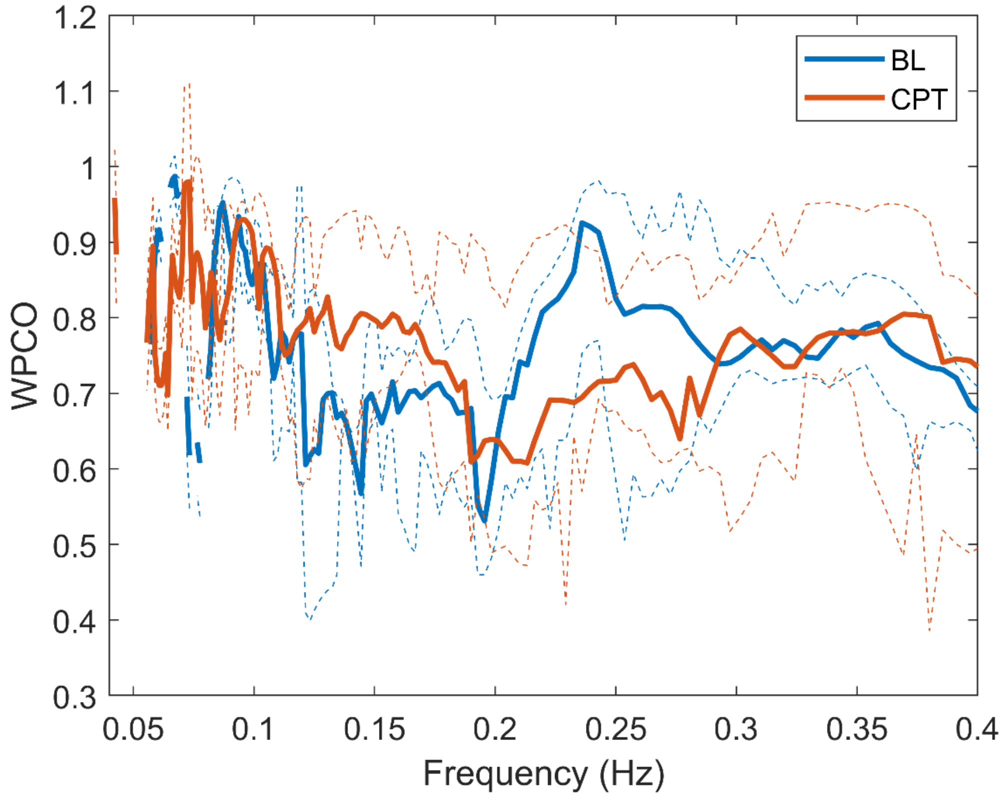

Figure S6. Wavelet phase coherence of RR interval and systolic blood pressure for the hypertensive pregnant group during baseline (BL) and cold pressor test (CPT) in terms of median and 95% IC. *Indicates significant differences between BL and CPT.

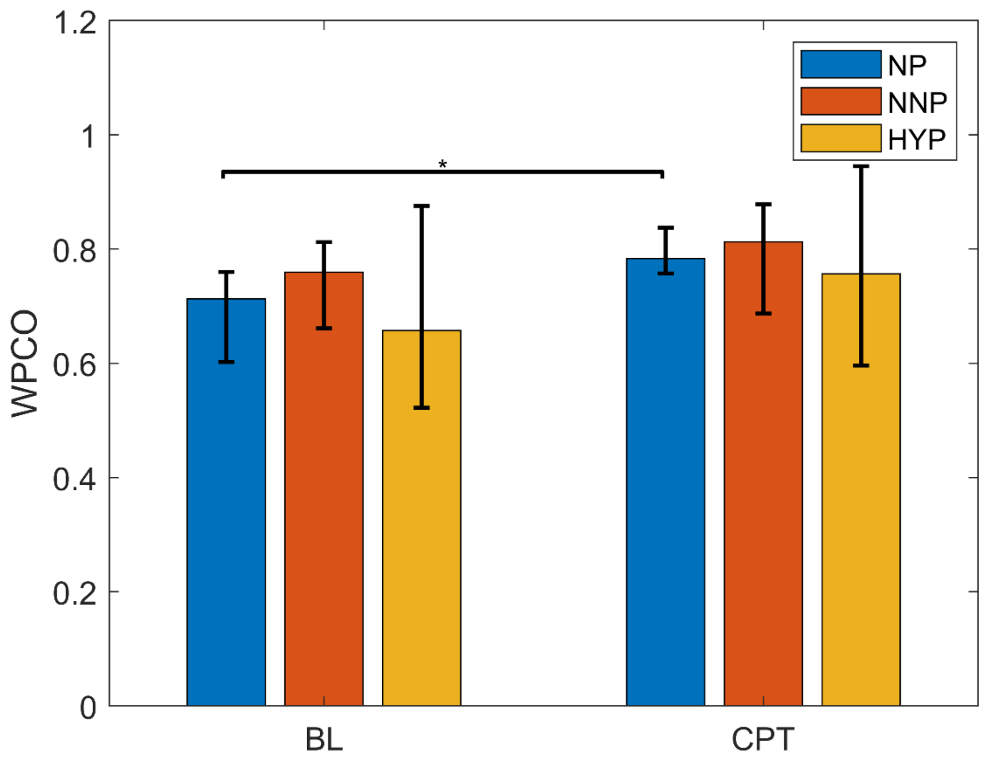

Figure S7. Wavelet phase coherence (WPCO) results for normotensive pregnant women (NP), normotensive non-pregnant women (NNP), and hypertensive pregnant women (HYP) in terms of median and 95% IC during baseline (BL) and cold pressor test (CPT). Results show WPCO between RR interval and DBP in the low-frequency band (LF). * represents p<0.05.

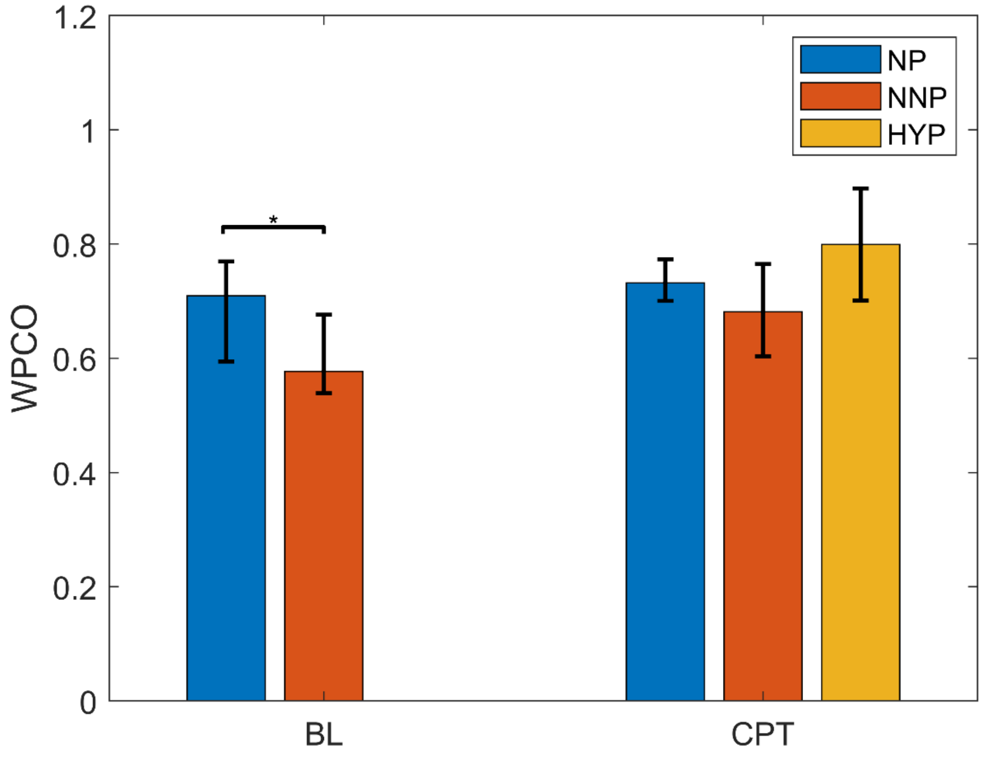

Figure S8. Wavelet phase coherence (WPCO) results for normotensive pregnant women (NP), normotensive non-pregnant women (NNP), and hypertensive pregnant women (HYP) in terms of median and 95% IC during baseline (BL) and Cold Pressor Test (CPT). Results show WPCO between respiration and diastolic blood pressure (DBP) in the low-frequency band (LF). No phase coherence values were obtained for the HYP group at baseline; therefore, the bar for this group is not shown. * represents p<0.05.

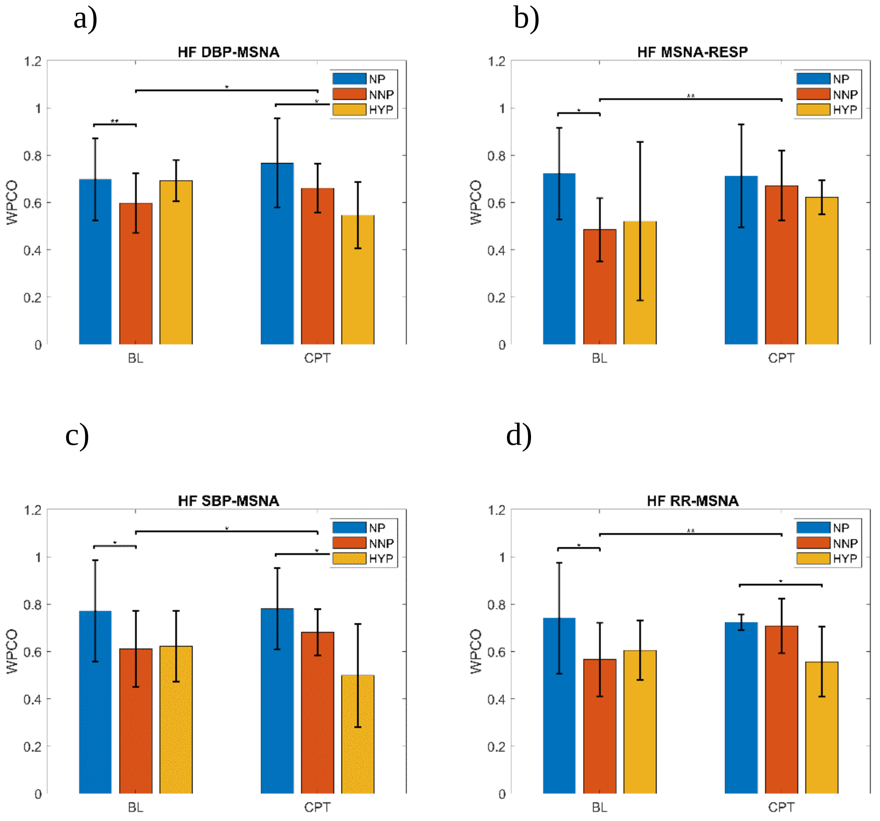

Figure S9. Wavelet phase coherence (WPCO) results for normotensive pregnant women (NP), normotensive non-pregnant women (NNP), and hypertensive pregnant women (HYP) in terms of median and interquartile range during baseline (BL) and cold pressor test (CPT). Results show WPCO between muscle sympathetic nerve activity (MSNA) and diastolic blood pressure (DBP) (a), MSNA and respiratory wave (RESP) (b), MSNA and systolic blood pressure (SBP) (c), and MSNA and RR interval (d) in the high-frequency band (HF). * represents $p<0.05$. ** represents $p<0.01$.

			RR-SBP	RR-DBP	RR-MSNA	RR-RESP	SBP-MSNA	SBP-RESP	DBP-MSNA	DBP-RESP	MSNA-RESP
**NP**	**BL**	HF	10	10	9	10	10	10	10	10	9
		LF	8	10	7	6	8	5	7	5	5
	**CPT**	HF	9	9	8	10	9	9	10	9	9
		LF	9	7	8	8	8	7	8	7	7
**NNP**	**BL**	HF	10	10	10	10	10	9	10	9	9
		LF	10	10	9	8	8	5	10	6	5
	**CPT**	HF	10	9	9	10	9	10	10	9	7
		LF	9	9	9	6	8	4	8	4	4
**HYP**	**BL**	HF	9	6	7	9	6	6	7	8	6
		LF	7	4	5	3	4	1	5	0	1
	**CPT**	HF	9	7	5	8	4	7	5	7	5
		LF	8	4	2	2	3	5	4	2	5

Table S1. Number of subjects per group with significant values of wavelet phase coherence results in the low and high-frequency band during the Baseline and Cold Pressor Test (CPT). The table shows the results for normotensive non-pregnant women (NNP), normotensive pregnant women (NP), and hypertensive pregnant women (HYP). RR: RR interval. DBP: Diastolic blood pressure. SBP: Systolic blood pressure. MSNA: Muscle sympathetic nerve activity. RESP: Respiratory wave.

	Baseline			Cold pressor test		
	NP(%)	NNP(%)	HYP(%)	NP(%)	NNP(%)	HYP(%)
**RR-SBP**	0	0	14.3	0	0	0
**RR-DBP**	0	0	0	0	0	0
**RR-MSNA**	0	0	0	25	0	0
**RR-RESP**	0	0	0	0	0	0
**SBP-MSNA**	0	0	0	0	0	0
**SBP-RESP**	0	0	0	0	0	0
**DBP-MSNA**	0	0	16.7	0	0	0
**DBP-RESP**	0	0	0	0	0	0
**MSNA-RESP**	0	0	0	0	0	0

Table S2. Percentage of outliers in phase coherence results for normotensive pregnant women (NP), normotensive-non pregnant women (NNP), and hypertensive pregnant women (HYP) during baseline and cold pressor test in the high-frequency band. Results are shown in terms of the percentage of elements more than 1.5 interquartile ranges above the upper quartile (75 percent) or below the lower quartile (25 percent).

	Baseline			Cold pressor test		
	NP(%)	NNP(%)	HYP(%)	NP(%)	NNP(%)	HYP(%)
**RR-SBP**	0	0	0	0	11.1	0
**RR-DBP**	20	0	0	14.3	0	0
**RR-MSNA**	0	11.1	0	0	0	0
**RR-RESP**	0	0	0	0	0	0
**SBP-MSNA**	0	0	0	0	0	0
**SBP-RESP**	0	0	0	0	0	0
**DBP-MSNA**	0	0	0	12.5	12.5	0
**DBP-RESP**	0	0	-	28.6	0	0
**MSNA-RESP**	0	0	0	0	0	0

Table S3. Percentage of outliers in phase coherence results for normotensive pregnant women (NP), normotensive-non pregnant women (NNP) and hypertensive pregnant women (HYP) during baseline and cold pressor test in the low-frequency band. Results are shown in terms of the percentage of elements more than 1.5 interquartile ranges above the upper quartile (75 percent) or below the lower quartile (25 percent).

		RR-SBP	RR-DBP	RR-MSNA	RR-RESP	SBP-MSNA	SBP-RESP	DBP-MSNA	DBP-RESP	MSNA-RESP
NP	BL	0.76 (0.58-0.94)	0.77 (0.56-0.98)	0.74† (0.51-0.97)	0.77 (0.46-1.08)	0.77† (0.56-0.98)	0.73 (0.51-0.95)	0.70† (0.53-0.87)	0.73 (0.53-0.93)	0.72† (0.53-0.91)
	CPT	0.78 (0.61-0.95)	0.82 (0.69-0.95)	0.72 (0.69-0.75)	0.81 (0.59-1.03)	0.78 (0.61-0.95)	0.83 (0.76-0.9)	0.77 (0.58-0.96)	0.82 (0.56-1.08)	0.71 (0.49-0.93)
NNP	BL	0.75* (0.65-0.85)	0.73 (0.59-0.87)	0.57* (0.41-0.73)	0.63 (0.45-0.81)	0.61* (0.45-0.77)	0.68 (0.42-0.94)	0.60* (0.47-0.73)	0.58 (0.4-0.76)	0.48* (0.35-0.61)
	CPT	0.83 (0.68-0.98)	0.81 (0.66-0.96)	0.71⅄ (0.59-0.83)	0.71 (0.53-0.89)	0.68⅄ (0.58-0.78)	0.73 (0.52-0.94)	0.66⅄ (0.56-0.76)	0.73 (0.43-1.03)	0.67 (0.52-0.82)
HYP	BL	0.72 (0.64-0.8)	0.78 (0.66-0.9)	0.61 (0.48-0.74)	0.59 (0.43-0.75)	0.62 (0.47-0.77)	0.70 (0.55-0.85)	0.69 (0.6-0.78)	0.61 (0.39-0.83)	0.52 (0.19-0.85)
	CPT	0.67 (0.49-0.85)	0.75 (0.54-0.96)	0.56 (0.41-0.71)	0.66 (0.44-0.88)	0.50 (0.28-0.72)	0.63 (0.41-0.85)	0.55 (0.41-0.69)	0.58 (0.37-0.79)	0.62 (0.55-0.69)

Table S4. Wavelet phase coherence results in the high-frequency band. The table shows the results for normotensive non-pregnant women (NNP), normotensive pregnant women (NP), and hypertensive pregnant women (HYP) during baseline (BL) and cold pressor test (CPT) in terms of median and interquartile range. RR: RR interval. DBP: Diastolic blood pressure. SBP: Systolic blood pressure. MSNA: Muscle sympathetic nerve activity. RESP: Respiratory wave. *: significant difference with CPT. †: significant difference with NNP. ⅄: Significant difference with HYP.

		RR-SBP	RR-DBP	RR-MSNA	RR-RESP	SBP-MSNA	SBP-RESP	DBP-MSNA	DBP-RESP	MSNA-RESP
NP	BL	0.68 (0.5-0.86)	0.71* (0.62-0.8)	0.68 (0.54-0.82)	0.77 (0.59-0.95)	0.68 (0.52-0.84)	0.68 (0.52-0.84)	0.76 (0.69-0.83)	0.71† (0.57-0.85)	0.65 (0.5-0.8)
	CPT	0.74 (0.59-0.89)	0.78 (0.71-0.85)	0.76 (0.59-0.93)	0.73 (0.6-0.86)	0.76 (0.59-0.93)	0.7 (0.58-0.82)	0.79 (0.71-0.87)	0.73 (0.66-0.8)	0.61 (0.44-0.78)
NNP	BL	0.73 (0.53-0.93)	0.76 (0.61-0.91)	0.74 (0.59-0.89)	0.69 (0.51-0.87)	0.78 (0.6-0.96)	0.7 (0.52-0.88)	0.76 (0.6-0.92)	0.58 (0.48-0.68)	0.64 (0.49-0.79)
	CPT	0.82 (0.7-0.94)	0.81 (0.69-0.93)	0.77 (0.59-0.95)	0.8 (0.62-0.98)	0.78 (0.64-0.92)	0.76 (0.55-0.97)	0.8 (0.69-0.91)	0.68 (0.57-0.79)	0.72 (0.55-0.89)
HYP	BL	0.65 (0.5-0.8)	0.66 (0.4-0.92)	0.62 (0.52-0.72)	0.61 (0.59-0.63)	0.56 (0.45-0.67)	0 (0-0)	0.63 (0.46-0.8)	0 (0-0)	0 (0-0)
	CPT	0.76 (0.62-0.9)	0.76 (0.54-0.98)	0.69 (0.55-0.83)	0.86 (0.68-1.04)	0.65 (0.44-0.86)	0.73 (0.56-0.9)	0.69 (0.59-0.79)	0.80 (0.6-1)	0.70 (0.49-0.91)

Table S5. Wavelet phase coherence results in the low-frequency band. The table shows the results for normotensive non-pregnant women (NNP), normotensive pregnant women (NP), and hypertensive pregnant women (HYP) during baseline (BL) and cold pressor test (CPT) in terms of median and interquartile range. RR: RR interval. DBP: Diastolic blood pressure. SBP: Systolic blood pressure. MSNA: Muscle sympathetic nerve activity. RESP: Respiratory wave. *: significant difference with CPT. †: significant difference with NNP.
